# Ectopic calcification and formation of mineralo-organic particles in arteries of diabetic subjects

**DOI:** 10.1038/s41598-020-65276-7

**Published:** 2020-05-22

**Authors:** Cheng-Yeu Wu, Jan Martel, John D. Young

**Affiliations:** 1 Laboratory of Nanomaterials, Chang Gung University, Taoyuan, 33302 Taiwan; 2 Center for Molecular and Clinical Immunology, Chang Gung University, Taoyuan, 33302 Taiwan; 3 Research Center of Bacterial Pathogenesis, Chang Gung University, Taoyuan, 33302 Taiwan; 4 Chang Gung Immunology Consortium, Linkou Chang Gung Memorial Hospital, Taoyuan, 33305 Taiwan; 5 Biochemical Engineering Research Center, Ming Chi University of Technology, New Taipei City, 24301 Taiwan

**Keywords:** Blood proteins, Predictive markers

## Abstract

Vascular calcification occurs in various diseases including atherosclerosis, chronic kidney disease and type 2 diabetes but the mechanism underlying mineral deposition remains incompletely understood. Here we examined lower limb arteries of type 2 diabetes subjects for the presence of ectopic calcification and mineral particles using histology, electron microscopy and spectroscopy analyses. While arteries of healthy controls showed no calcification following von Kossa staining, arteries from 83% of diabetic individuals examined (19/23) revealed microscopic mineral deposits, mainly within the tunica media. Mineralo-organic particles containing calcium phosphate and proteins such as albumin, fetuin-A and apolipoprotein-A1 were detected in calcified arteries. Ectopic calcification and mineralo-organic particles were observed in a majority of diabetic patients and predominantly in arteries showing hyperplasia. While a low number of subjects was examined and information about disease severity and patient characteristics is lacking, these calcifications and mineralo-organic particles may represent signs of tissue dysfunction.

## Introduction

The prevalence of diabetes worldwide reached 422 millions people in 2014, representing nearly a four-fold increase since 1980^1^. Type 2 diabetes is associated with obesity which affects 13% of the world adult population^2^. Diabetic subjects show increased cardiovascular mortality mainly due to a high prevalence of obesity, hypertension and dyslipidemia^3^. Vascular calcification is believed to contribute to cardiovascular disease as atherosclerotic individuals with calcified arteries show increased mortality risk compared to calcification-free individuals^4^.

Vascular calcification is considered an active process in which vascular smooth muscle cells (VSMCs) differentiate into osteoblast-like cells that deposit calcium phosphate in arteries^5^. Various mechanisms have been proposed to account for ectopic mineral deposition in the body. For instance, loss of calcification inhibitors such as matrix Gla protein and fetuin-A leads to ectopic mineralization in animal models^5^. Similarly, disruption of ion homeostasis due to chronic kidney disease can increase the concentration of calcium and phosphate in body fluids and contribute to mineral deposition in the arteries of human patients. Moreover, apoptosis of vascular smooth muscle cells is believed to lead to the release of apoptotic bodies and membrane vesicles which may provide nidi for the formation of larger mineral deposits^5^.

We observed earlier that kidney tissues from patients with chronic kidney disease or renal cancer harbor mineralo-organic nanoparticles (NPs) containing blood proteins that normally act as calcification inhibitors^6^. These mineral NPs form when the concentrations of calcium, carbonate and phosphate exceed saturation^7–9^ and they readily bind to various organic molecules found in body fluids^10–12^. Research performed by our group^13,14^ and others^15,16^ suggest that the mineral particles may represent precursors of mineral deposition in the body. Yet, more studies are needed to determine the characteristics, distribution and effects of these particles in the human body.

Previous studies have shown that radiography and computed tomography can be used to detect ectopic calcification in arteries of diabetic subjects^17,18^. In the present study, we used histology and a nanomaterial-based approach to examine the formation of mineralo-organic particles in human arteries of diabetic subjects. To our knowledge, these results are the first to indicate that lower limb arteries of individuals with type 2 diabetes harbor mineralo-organic particles similar to the particles described earlier in biological fluids.

## Methods

### Human tissue samples

The use of human samples was approved by the Institutional Review Board of Chang Gung Memorial Hospital (Linkou, Taiwan). The experiments were performed in accordance with the Declaration of Helsinki and the approved guidelines and regulations. Written informed consent was obtained from the subjects. Artery tissues were obtained from type 2 diabetes mellitus patients (n = 23). Tissues consisted of discarded lower limb arteries (~1 cm in length) removed during surgical amputation. Discarded lower limb arteries of healthy controls (n = 4) who underwent amputation following trauma were also examined. Each artery sample was divided for preparation of paraffin sections and ultrathin sections as described below.

### Tissue preparation

Blood vessel specimens were fixed in 10% formaldehyde and mounted on paraffin blocks which were sectioned using a microtome (Cambridge Scientific, HM350) to produce sections with a thickness of 4–5 μm.

### Hematoxylin and eosin staining

Histological staining was performed as before^6^. Briefly, for hematoxylin and eosin (H&E) staining, tissue sections were deposited on glass slides and incubated overnight in a laminar flow hood. Specimens were heated at 70 °C for 30 min. Sections were immersed into a fresh solution of xylene three times for 15 min to remove paraffin. Sections were rehydrated with 95%, 80% and 70% ethanol for 5 min each time. Rehydrated specimens were washed with double-distilled water (thereafter referred simply as “water”) for 5 min. Cell nuclei were stained with hematoxylin for 8 min, followed by washing in water. Specimens were dipped 10 times in ethanol (95%). Eosin Y was used as counterstain for 1 min. Dehydration was done in 95% and 100% ethanol, followed by treatment with xylene, for 10 min each time. Microscopy observations were done with a BX51 light microscope (Olympus) and a Flex digital camera (Diagnostic Instruments) as done before^6^.

### von Kossa staining

This procedure was performed as described previously^6^, with minor modifications. Briefly, deparaffinized and rehydrated blood vessel specimens were incubated with a solution of 5% silver nitrate. Samples were exposed to UV light for approximately 20 min, prior to washing in water. Staining was done with 5% sodium thiosulfate and nuclear fast red (Sigma-Aldrich) for 5 min each time. Dehydrated samples were examined by light microscopy as above. Some microscopy images were combined to form larger images using the PTGui Pro software (New House Internet Services, version 10.0.16).

### Scanning electron microscope and energy-dispersive X-ray spectroscopy

Paraffin sections of blood vessels were placed onto copper pieces and dried overnight in a laminar flow hood. Specimens were coated with gold using a sputter coater (E-1010, Hitachi Science Systems). Samples were observed under an S-3000N scanning electron microscope (SEM) (Hitachi Science Systems). Energy-dispersive X-ray spectroscopy (EDX) analysis was performed using an EMAX Energy EX-400 EDX device (Horiba). Samples were irradiated for 30 sec and data acquisition was performed in point mode using the EMAX software (Horiba).

### Transmission electron microscopy

Ultrathin sections were prepared as before^6^. Sections were placed onto carbon-coated formvar copper grids. Contrast staining was done with 2% uranyl acetate. Specimens were observed under a JEOL 1230 transmission emission microscope (TEM) operated at 100 kV and equipped with a Gatan Orius SC1000 digital camera (JEOL).

### Preparation of mineralo-organic particles

Mineralo-organic particles were prepared as described earlier^6,7^. Briefly, human serum (HS) was obtained from healthy volunteers using conventional venipuncture. Mineralo-organic particles were prepared by adding 3 mM CaCl_2_ and Na_2_HPO_4_ into DMEM (Gibco) containing 10% HS or fetal bovine serum (FBS) (PAA Laboratories), followed by incubation for one week in cell culture conditions (37 °C, 5% CO_2_, humidified air). Particles were pelleted by centrifugation at 16,000 × g for 15 min at 4 °C and washed twice with HEPES buffer (20 mM HEPES, 1 mM CaCl_2_, 2 mM Na_2_HPO_4_, 150 mM NaCl) using the same centrifugation procedure.

### Immunogold staining

Immunogold staining was done as described before^6,19^. Thin sections of blood vessel tissues were placed on carbon-coated formvar nickel grids and treated for 25 min with 1% fish gelatin (Sigma) in 0.1 M HEPES buffer (pH 8.0). Grids were incubated with a primary antibody: anti-human serum fetuin-A (α-HSF, 1:50, Santa Cruz Biotechnology, sc-133146), anti-human serum albumin (α-HSA, 1:30, Santa Cruz Biotechnology, sc-46293), anti-apolipoprotein-A1 (α-apo-A1, 1:30, Santa Cruz Biotechnology, sc-376818), anti-HS-NPs (α-HS-NPs, 1:60; prepared as before^20^), or anti-fengycin B (α-FenB, 1:50; described before^21^). Incubated sections were rinsed with HEPES buffer for 15 min. Rinsed sections were blocked with 1% fish gelatin in HEPES buffer for 20 min. Specimens were treated with secondary 5-nm-gold-conjugated goat anti-rabbit antibody (α-HSF, Cytodiagnostics, AC-30-02-05; α-HSA, Cytodiagnostics, AC-10-11-05; α-apo-A1, BBI Solutions, EM.GMHL10; α-HS-NP, BBI Solutions, EM.GAR10; α-FenB, BBI Solutions, EM.GAR10) for 1 h. TEM observations were performed as above.

### Immunofluorescence

Tissue sections were heated at 70 °C for 30 min. Tissues were immersed in xylene, rehydrated in increasing concentration of ethanol, and incubated in PBS (pH 7.4). Specimens were incubated in antigen retrieval buffer (10 mM Tris-HCl, 1 mM EDTA, 0.05% Tween 20, pH 10) in a microwave (560 W) for 6 min. Samples were equilibrated at room temperature and immersed in 1% H_2_O_2_ for 5 min. Tissue sections were incubated in blocking solution (2% fish gelatin in PBS) for 2 h at room temperature, and washed with 0.5% Tween 20 in PBS (PBST) for 5 min, three times. Sections were incubated overnight with the fluorescent conjugated polyclonal antibody α-HSF (Bioss Antibodies, bs-2922R-A594) or α-HSA (Dako, F0117) at a dilution of 1:200 and 1:100, respectively. After washing, sections were mounted using mounting medium containing DAPI (Santa Cruz Biotechnology, UltraCrus Aqueous Mounting Medium, sc-24941) prior to observation under a DM6B fluorescence microscope (Leica) or LSM780 confocal fluorescence microscope (Zeiss). Images were processed and analyzed using the Las X software (Leica).

## Results

**Figure 1:**
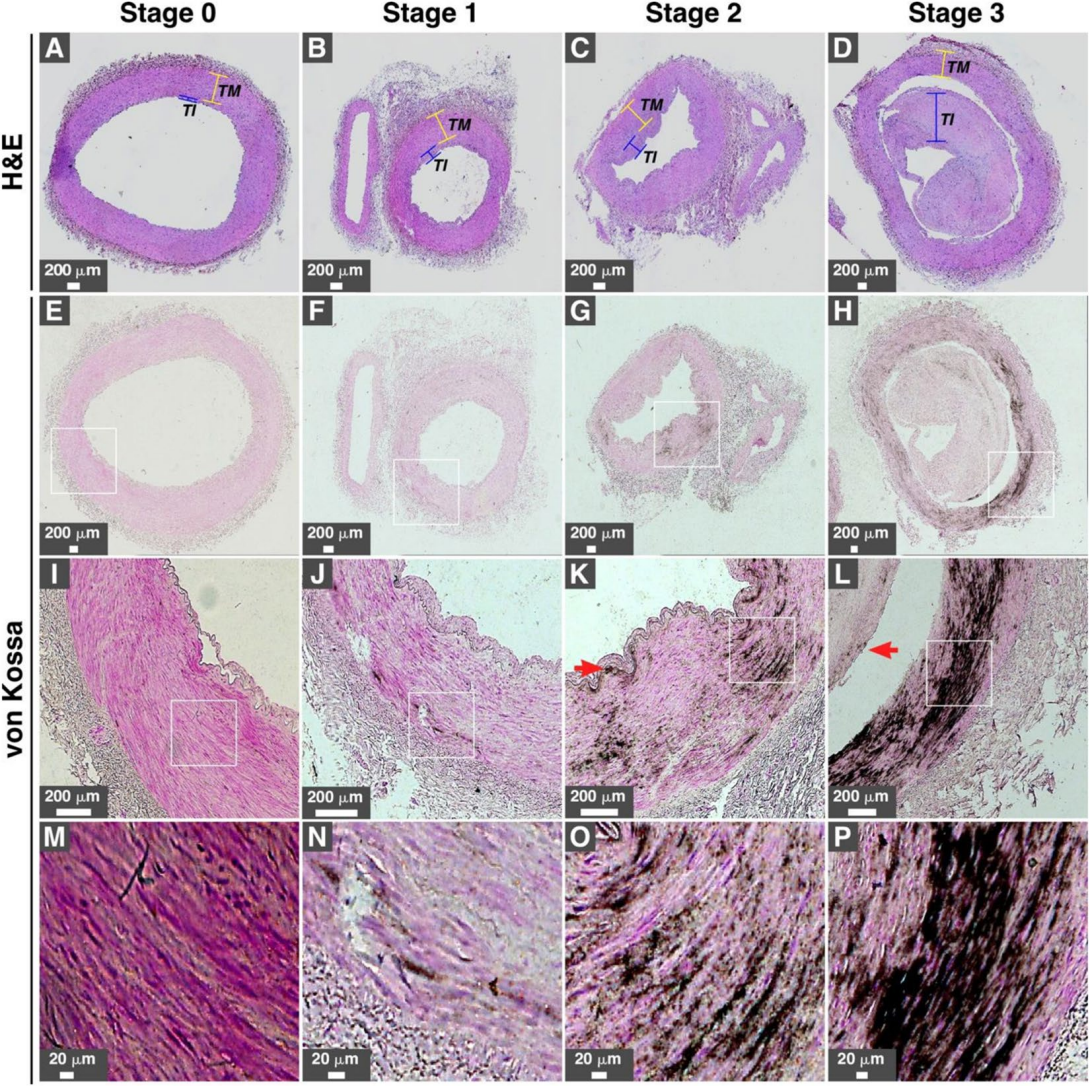
Calcification of lower limb arteries in type 2 diabetes subjects. (**A–D**) H&E staining and (**E–P**) von Kossa staining of lower limb arteries from diabetic individuals (n = 23) were processed as described in the *Methods*. Intimal thickening was observed in (**B–D**), while the intima in A was normal. White rectangles represent the magnified area observed in the images found in the row below. Calcification level was graded into four stages based on the abundance and location of black mineral precipitates; stage 0: absence of black mineral deposit; stage 1: low amount of black precipitates; stage 2: abundant black mineral plaques extending into the layers of the media; stage 3: extensive black mineral deposition, including in the tunica media and surrounding the whole artery. Red arrows in K and L indicate the presence of intimal calcification. Table 1 shows the information about media calcification level, intimal thickening, and intimal calcification noted for the collected artery specimens examined in the present study. *TI*, tunica intima; *TM*, tunica media.

We examined lower limb artery tissues from 23 diabetic subjects for the presence of vascular calcification. For comparison, we also examined lower limb arteries of four healthy controls. Hematoxylin and eosin (H&E) staining of arteries from healthy controls showed normal histological features and no mineral precipitate was observed following von Kossa staining (Table 1 and Supplementary Fig. S1). Arteries from diabetic subjects showed subendothelial hyperplasia or intimal thickening in 75% of cases (Fig. 1, Table 1, and Supplementary Table S1). Based on von Kossa staining and optical microscopy, we observed that specimens from 19 out of 23 diabetic subjects (83%) contained calcification (Table 1; representative images of calcified tissues are shown in Fig. 1). Calcification level was classified into four stages (Table 1): absence of calcification (stage 0); fine mineral granulations (stage 1); mineral deposits with plaques extending in the layers of the media tunica (stage 2); and extensive mineral deposits involving the entire circumference of the artery (stage 3). Representative images for each calcification stage are shown in Fig. 1.

**Table 1:** Calcification level of lower limb arteries from type 2 diabetic subjects.

Calcification Level	Healthy Controls (n = 4)	Diabetic Subjects (n = 23)			
	Stage 0	Stage 0	Stage 1	Stage 2	Stage 3
	4	4	3	6	10
Intimal Thickening	–	–	1	6	10
Intimal Calcification	–	–	–	3	5

Calcification staging of lower limb arteries from healthy controls (n = 4) and diabetic subjects (n = 23) was based on the detection and abundance of black precipitates in the tunica media following von Kossa staining and observation under optical microscopy as described in the *Methods*. Calcification level was based on four stages; stage 0: absence of black mineral deposit; stage 1: detection of black granules; stage 2: abundant black mineral plaques with some extending in the layer of the media; stage 3: extensive black mineral deposition including in the tunica media and surrounding the whole artery. Intimal thickening was assessed by observation of H&E-stained tissues. Specimens with intima over 0.2 mm and containing multiple cell layers were classified as positive for intimal thickening.

**Figure 2:**
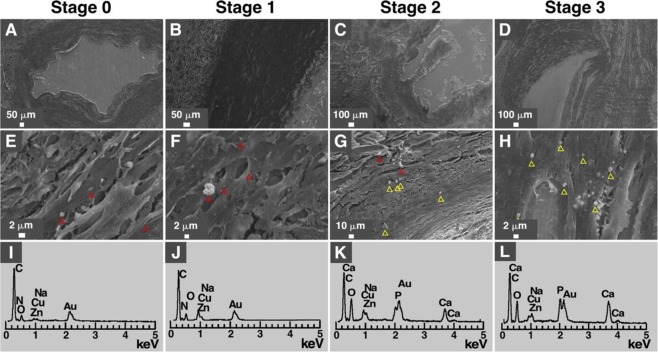
Energy-dispersive X-ray spectroscopy analysis of human calcified arteries of diabetic subjects. (**A–H**) SEM images and (**I–L**) EDX analysis of lower limb arteries of diabetic individuals. No particles were observed at low magnification (**A–D)**; however, particles were detected at higher magnification (**E–H**). Red triangles: granules that showed no signal for either calcium or phosphorus. Yellow triangles: granules that showed signals for calcium, oxygen and phosphorus, consistent with the presence of calcium phosphate in these particles. Gold (Au) originated from sputter coating.

We used SEM to examine the morphology and ultrastructure of the calcified areas in arteries of diabetic individuals (Fig. 2). High magnification observations revealed the presence of mineral granules with sub-micron size in artery specimens corresponding to all stages (Fig. 2E–H). Low amounts of particles were detected in stages 0 and 1 based on SEM observations (Fig. 2E,H) but these particles did not contain calcium or phosphorus based on EDX analysis (Fig. 2I,J). In contrast, the mineral particles observed in samples from stages 2 and 3 (Fig. 2G,H) contained calcium and phosphorus (Table 2 and Fig. 2K,L; the EDX spectra in K and L correspond to the particles highlighted in yellow in G and H, respectively). A few NPs were observed in the arteries of healthy controls under SEM but these particles did not contain calcium and phosphorus when analyzed with EDX (Supplementary Fig. S1).

**Table 2:** Chemical elements detected using EDX in mineral particles found in lower limb arteries of diabetic patients.

Calcification stage	Specimen	Number of particles analyzed	Elements identified				Ca/P ratio
			Ca	P	Ca, P	Ca, P, Mg	
Stage 0	21730166-1	4	1	0	0	0	–
Stage 1	20292788-2	24	2	0	0	0	–
	21757540-1	26	6	1	1	0	1.6
Stage 2	3007510-2	24	10	0	9	5	0.88 ± 0.13
	10005751-3	22	10	0	8	4	1.11 ± 0.17
Stage 3	21514588-1	25	0	0	20	2	1.53 ± 0.35
	2473506-1	15	2	0	4	7	1.59 ± 0.07
	21732031-1	39	1	0	32	4	1.61 ± 0.02

Calcification level was assessed as described in the legend of Table 1. Chemical elements in the media tunica of arteries were determined using EDX analysis. “Ca, P” refers to the number of particles in which both calcium and phosphorus were detected, which allowed determination of “Ca/P ratios”.

**Figure 3:**
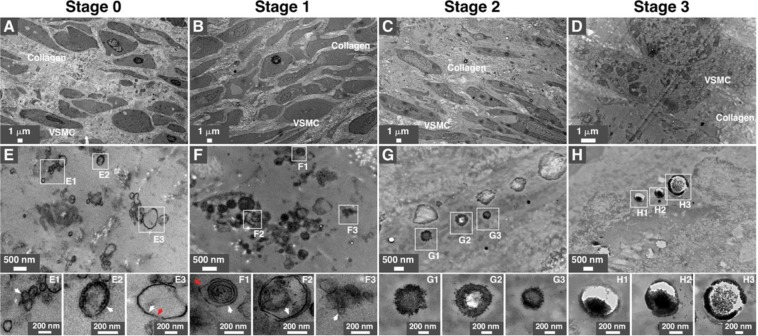
Transmission electron microscopy observations of mineral particles in arteries of diabetic subjects. Artery tissues were prepared and observed under thin-section TEM as described in *Methods*. Low (**A–D**) and high (**E–H**) magnification images of tunica media are shown. Vesicles-like particles are denoted by arrows, with white arrows indicating single membranes and red arrows denoting double membranes. White rectangles indicate enlarged areas of the insets. VSMC, vascular smooth muscle cells.

TEM observations also revealed the presence of mineral particles within the artery samples of all stages (Fig. 3). Particles in stages 0 and 1 appeared to be delineated by a lipid membrane (Fig. 3E,F, see insets for higher magnification). Particles with a multilayer structure consisting of alternating electron-dense and electron-lucid layers were observed in all stages (Fig. 3, see insets). These multilayer mineral particles are similar to the particles observed earlier in biological fluids^7^ and renal tissues^6^.

**Figure 4:**
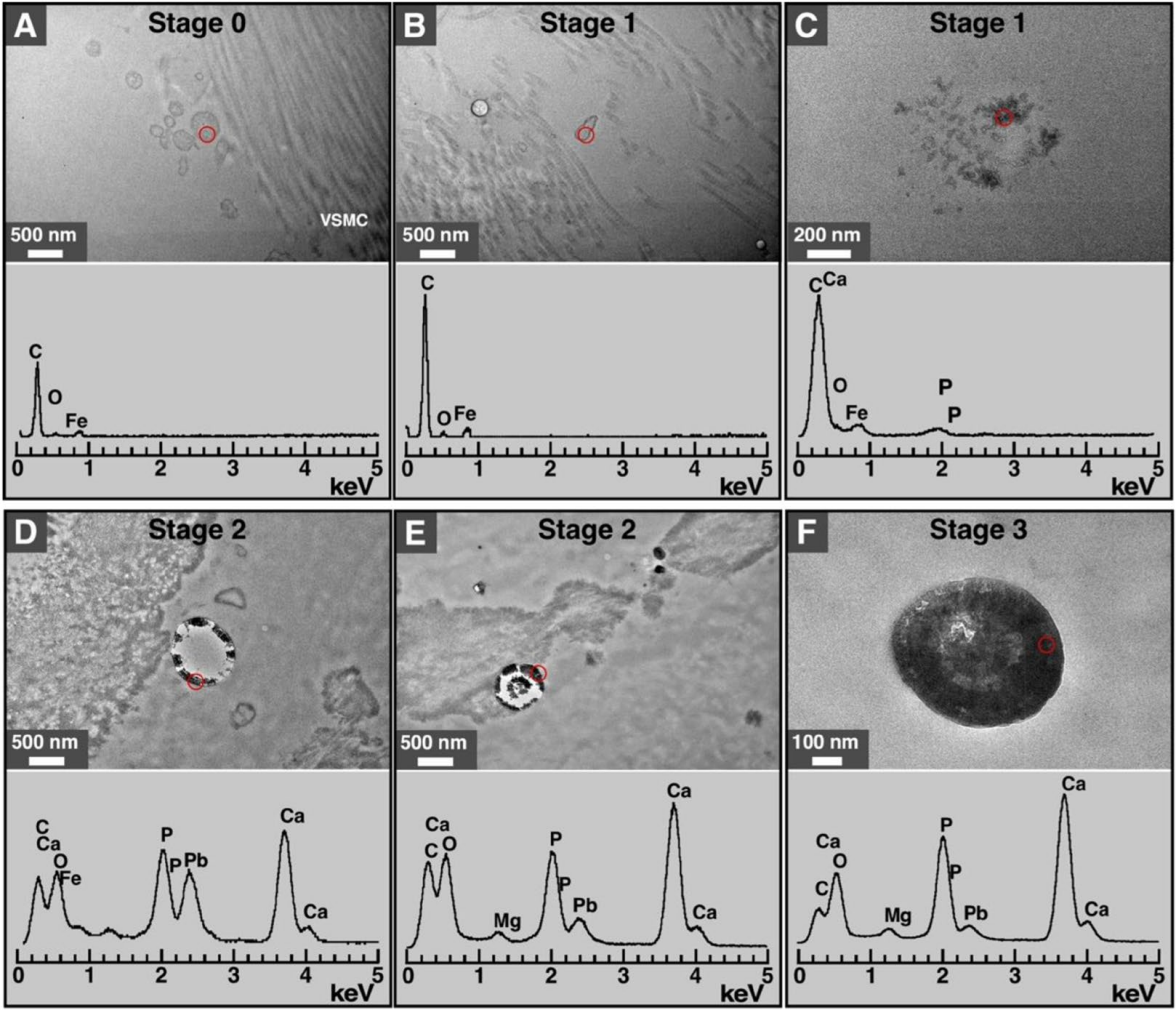
EDX analysis of vesicles and mineral particles in artery tissues of diabetic subjects. TEM and EDX analysis revealed the presence of vesicle structures containing no calcium or phosphorus in specimens corresponding to stages 0 and 1 (**A**,**B**). Some vesicle structures from stage 1 specimens contained low amounts of calcium and phosphorus (**C**). Mineral particles containing calcium and phosphorus were observed in specimens from stages 2 and 3 (**D**–**F**). Red circles indicate the areas that were scanned for EDX. Iron (Fe) and lead (Pb) may have originated from the reagents used during sample preparation.

We used TEM and EDX analysis to examine the vesicles that appeared to be delineated by a lipid membrane (Fig. 4). While the vesicles observed in specimens from stages 0 and 1 showed no calcium or phosphorus (Fig. 4A,B), some mineral deposits found in these samples showed peaks of carbon and phosphorus (Fig. 4C). Vesicles detected in specimens from stages 2 and 3 showed calcium and phosphorus peaks (Fig. 4D–F), consistent with the presence of calcium phosphate within these vesicles.

**Figure 5:**
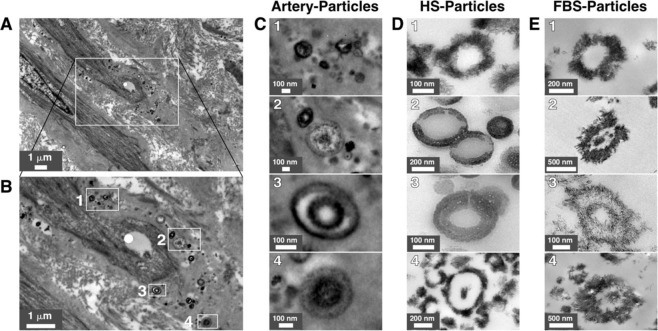
Morphology of mineralo-organic particles observed in lower limb artery tissues from diabetic patients. (**A–C**) Mineral particles observed in artery tissues by thin-section TEM. (**D**,**E**) Mineralo-organic particles labeled as HS-particles (**D**) or FBS-particles (**E**) were prepared as described in the *Methods*. The particles observed in arteries showed morphologies and sizes similar to that of prepared mineralo-organic particles. FBS, fetal bovine serum; HS, human serum.

We compared the morphologies and composition of artery-derived mineral particles with mineralo-organic particles prepared in human serum (HS-Particles) or fetal bovine serum (FBS-Particles), which have been found earlier to mimic the mineral particles detected in body fluids^7,22^. Under TEM, the prepared particles showed a round morphology and a size distribution (~50 nm to 1.5 μm) similar to that of the particles detected in calcified arteries from diabetic individuals (Fig. 5). The round morphology of the mineralo-organic particles was attributed earlier to stabilization of amorphous calcium phosphate phase by organic components such as proteins found in biological fluids^7,8,14^.

**Figure 6:**
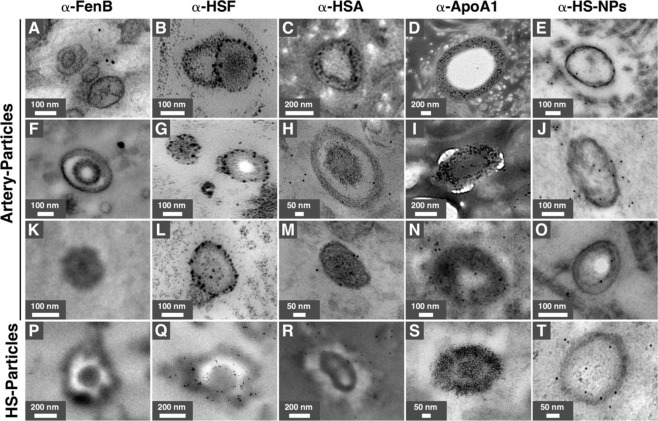
Immunogold staining of mineral particles observed in human arteries of diabetic subjects and specimens prepared *in vitro*. Human artery tissues (**A–O**) and HS-particles prepared *in vitro* (**P–T**) and processed for thin-section TEM and immunogold labeling as described in the *Methods*. Gold-NP-coupled antibodies used for these experiments are indicated on top. An antibody that reacts against FenB, a protein from *Bacillus subtilis*, was used as a negative control. ApoA1, apolipoprotein-A1; FenB, fengycin B; HSA, human serum albumin; HSF, human serum fetuin-A; HS-NPs, human serum-nanoparticles.

In order to confirm the presence of proteins in artery particles, we used immunogold staining coupled with TEM. Our results showed that antibodies that react against HSF, HSA, and apo-A1 bound to artery particles as shown by the presence of gold particles in association with the external or internal layers (Fig. 6). A negative control antibody that reacts against fengycin B (α-FenB), a bacterial protein from *Bacillus subtilis*, failed to bind to artery particles (Fig. 6A,F,K). We also used an antibody characterized earlier as binding to various proteins from human serum (α-HS-NPs). As expected, this antibody labeled all artery particles (Fig. 6E,J,O). Similar results were obtained for particles prepared in HS and used as a control for comparison (Fig. 6P–T).

**Figure 7:**
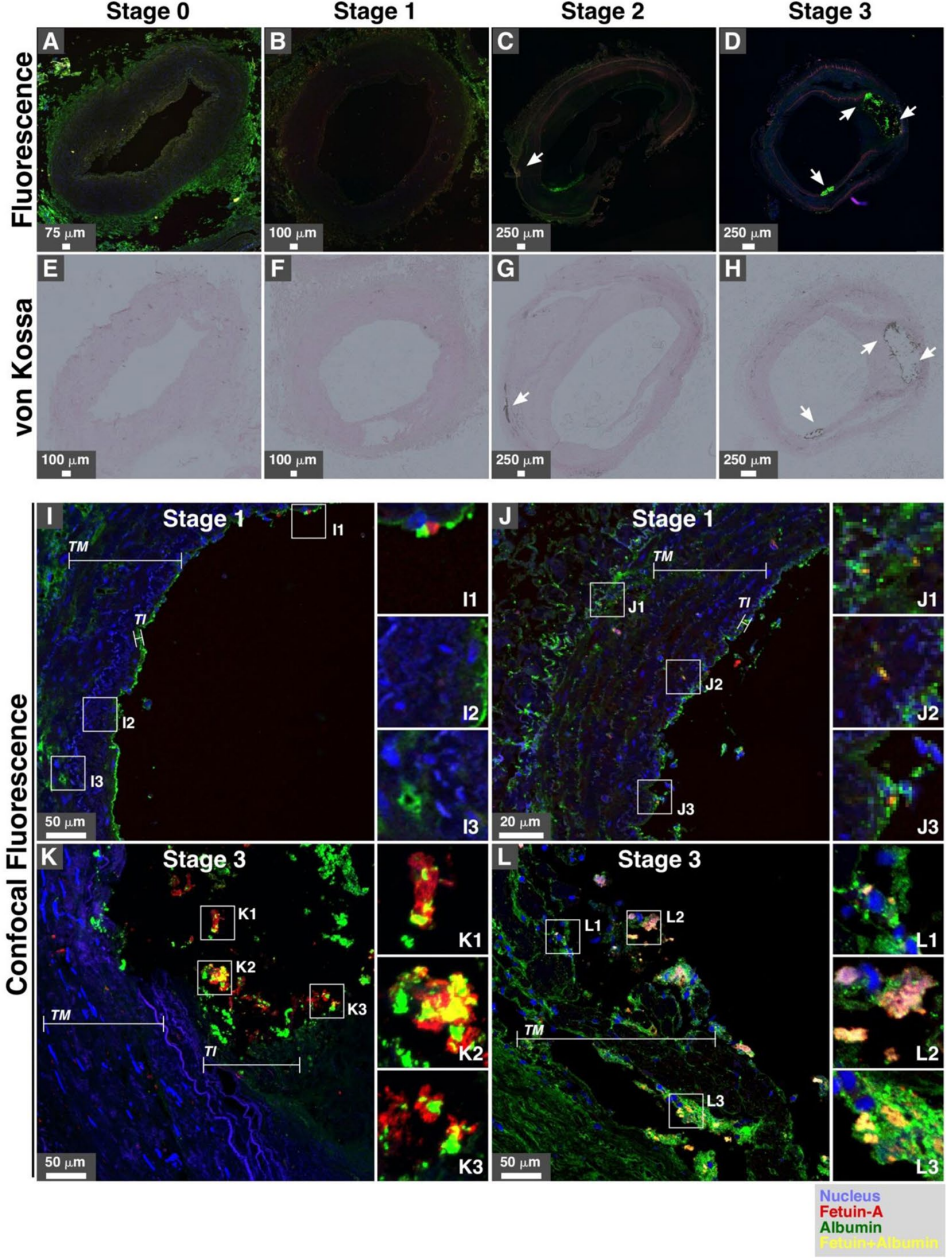
Immunohistological staining of artery tissues from diabetic subjects showing colocalization of serum proteins and mineral deposits. Serial tissue sections were processed for albumin and fetuin-A immunofluorescence staining (**A–D**), von Kossa staining (**E–H**), and confocal fluorescence (**I–L**) as described in the *Methods*. White arrows indicate positive signals for immunofluorescence (albumin and/or fetuin-A) and von Kossa staining (calcification). White rectangles indicate the enlarged areas of the insets. *TI*, tunica intima; *TM*, tunica media.

We also performed immunofluorescence staining of artery tissues using antibodies that react against serum proteins. Our results showed that both HSF and HSA were enriched in calcified arteries belonging to stages 2 and 3, whereas minimal fluorescence was noted in specimens from stages 0 and 1 (Fig. 7, arrows in C, D, G, and H show fluorescence staining and calcification based on von Kossa staining). HSF and HSA colocalized and formed small particles in artery tissues examined using confocal fluorescence microscopy (Fig. 7I–L). While specimens of stage 1 showed minor colocalization of HSA and HSF (Fig. 7I,J), specimens from stage 3 showed more extensive colocalization (Fig. 7K,L). Some particles containing HSF and HSA were also in close proximity with nucleic acids (Fig. 7, see insets).

## Discussion

We observed that the majority of artery tissues from diabetic patients contain macroscopic calcification as well as mineralo-organic particles. TEM observations indicated that artery tissues contained lipid membrane vesicles, and the presence of calcium and phosphorus (and thus possibly phosphate) within the particles was associated with a higher propensity for ectopic calcification. A possible interpretation for this observation is that the membrane vesicles may represent the nucleating agents that induce the formation of mineral particles in calcified artery tissues. The mineral particles are thus likely to represent precursors of ectopic calcification in this context. Consistent with this possibility, we observed earlier that membrane vesicles derived from serum can nucleate mineralo-organic NPs in biological fluids^20^.

Our findings are consistent with the observations made by other groups. For instance, Price *et al*. described the formation of fetuin-mineral complexes in the serum of rats treated with the bisphosphonate etidronate^23^ or vitamin D^24^. Jahnen-Dechent and colleagues described the formation of calciprotein particles (CPPs) in the ascites of a patient with calcifying peritonitis^25^. Primary CPPs consisted of small clusters of amorphous calcium phosphate and fetuin-A which gradually ripened with time to form more crystalline and larger secondary CPPs^25^. Matsui *et al*. observed that a precipitate of calciprotein particles formed in the serum of rats treated with adenine to induce kidney failure^26^. Schlieper *et al*. described mineral particles in iliac arteries of human subjects with chronic kidney disease requiring dialysis^15^. This group identified apoptotic bodies or matrix vesicles as possible nucleating agents for the minerals^15^. Yamada and colleagues identified CPPs in the serum of 10 diabetic patients and observed that their levels increased after meal intake and in subjects with reduced renal functions^27^.

The main limitations of this exploratory study include the low number of subjects and the absence of information regarding disease severity or the gender and age of the patients studied. Given that the specimens were obtained from diabetic subjects who required amputation, the samples probably reflect a late disease stage. While we observed mineralo-organic particles in artery tissues from these diabetic subjects, the clinical significance of vascular deposition and the presence of mineral particles in this context will require further studies. Recent studies indicate that vascular calcification is associated with increased mortality risk in patients with chronic kidney disease^28,29^. The presence of mature calciprotein particles was also associated with hypertension or chronic kidney disease^30^. Similarly, artery calcification—especially in the iliac arteries but also in the aorta and carotid arteries—was associated with increased total and cardiovascular mortality in human patients^31^. Medial calcification was also identified as a strong predictor of cardiovascular mortality in type 2 diabetes patients^4^. These results suggest that the presence of mineral NPs in human blood may be used as a marker of calcification status and disease progression. Detection of calciprotein particles in body fluids represent an interesting approach in this context^32,33^.

We observed earlier that various minerals that form in the presence of organic compounds will form an amorphous phase with rounded, cell-like morphologies before transforming into crystalline mineral particles^7,34^. These mineralo-organic NPs activate caspase-1 and NLRP3 inflammasome in macrophages and may therefore induce inflammation^22^. Similarly, the particles activated neutrophils and the release by these cells of neutrophil extracellular traps (NETs) which further activated bystander macophages via high-mobility group protein B1 (HMGB1)^35^. The possibility that these particles may contribute to inflammation in humans remains to be examined.

The approach used here may be used to study the formation and role of mineral particles in the human body. Inhibition of the formation of mineral particles or their dissolution may be beneficial in diabetic patients or in subjects with chronic disease. Further studies are needed to examine these possibilities.

## Supplementary Information

Supplementary Information.

## References

[1] WHO. *Diabetes (fact sheets)*, http://www.who.int/news-room/fact-sheets/detail/diabetes (2017).

[2] WHO. *Obesity and overweight*, http://www.who.int/en/news-room/fact-sheets/detail/obesity-and-overweight (2018).

[3] Leon BM, Maddox TM (2015). Diabetes and cardiovascular disease: Epidemiology, biological mechanisms, treatment recommendations and future research. World J. Diabetes.

[4] Chen NX, Moe SM (2003). Arterial calcification in diabetes. Curr. Diab Rep..

[5] Shroff RC, Shanahan CM (2007). The vascular biology of calcification. Semin. Dial..

[6] Wong TY (2015). Detection and characterization of mineralo-organic nanoparticles in human kidneys. Sci. Rep..

[7] Young JD (2009). Putative nanobacteria represent physiological remnants and culture by-products of normal calcium homeostasis. PLoS one.

[8] Young JD (2009). Characterization of granulations of calcium and apatite in serum as pleomorphic mineralo-protein complexes and as precursors of putative nanobacteria. PLoS one.

[9] Wu CY, Martel J, Young D, Young JD (2009). Fetuin-A/albumin-mineral complexes resembling serum calcium granules and putative nanobacteria: demonstration of a dual inhibition-seeding concept. PLoS one.

[10] Martel J (2011). Comprehensive proteomic analysis of mineral nanoparticles derived from human body fluids and analyzed by liquid chromatography-tandem mass spectrometry. Anal. Biochem..

[11] Martel J (2016). Fatty acids and small organic compounds bind to mineralo-organic nanoparticles derived from human body fluids as revealed by metabolomic analysis. Nanoscale.

[12] Wu CY, Martel J, Young JD (2018). Comprehensive organic profiling of biological particles derived from blood. Sci. Rep..

[13] Martel J, Wu CY, Young JD (2016). Translocation of mineralo-organic nanoparticles from blood to urine: a new mechanism for the formation of kidney stones?. Nanomedicine.

[14] Martel J, Wu CY, Peng HH, Young JD (2018). Mineralo-organic nanoparticles in health and disease: an overview of recent findings. Nanomedicine.

[15] Schlieper G (2010). Ultrastructural analysis of vascular calcifications in uremia. J. Am. Soc. Nephrol..

[16] Schlieper G (2010). Analysis of calcifications in patients with coral reef aorta. Ann. Vasc. Surg..

[17] Rennenberg RJ, Schurgers LJ, Kroon AA, Stehouwer CD (2010). Arterial calcifications. J. Cell Mol. Med..

[18] Cardenas V, Seo K, Sheth S, Meyr AJ (2018). Prevalence of lower-extremity arterial calcification in patients with diabetes mellitus complicated by foot disease at an urban US tertiary-care center. J. Am. Podiatr. Med. Assoc..

[19] Wong TY (2015). Nanoparticle conversion to biofilms: *in vitro* demonstration using serum-derived mineralo-organic nanoparticles. Nanomedicine.

[20] Wu CY (2013). Membrane vesicles nucleate mineralo-organic nanoparticles and induce carbonate apatite precipitation in human body fluids. J. Biol. Chem..

[21] Wu CY (2007). Nonribosomal synthesis of fengycin on an enzyme complex formed by fengycin synthetases. J. Biol. Chem..

[22] Peng HH (2013). Physicochemical and biological properties of biomimetic mineralo-protein nanoparticles formed spontaneously in biological fluids. Small.

[23] Price PA (2002). Discovery of a high molecular weight complex of calcium, phosphate, fetuin, and matrix gamma-carboxyglutamic acid protein in the serum of etidronate-treated rats. J. Biol. Chem..

[24] Price PA, Williamson MK, Nguyen TM, Than TN (2004). Serum levels of the fetuin-mineral complex correlate with artery calcification in the rat. J. Biol. Chem..

[25] Heiss A (2008). Hierarchical role of fetuin-A and acidic serum proteins in the formation and stabilization of calcium phosphate particles. J. Biol. Chem..

[26] Matsui I (2009). Fully phosphorylated fetuin-A forms a mineral complex in the serum of rats with adenine-induced renal failure. Kidney Int..

[27] Yamada H (2018). Daily variability in serum levels of calciprotein particles and their association with mineral metabolism parameters: A cross-sectional pilot study. Nephrology.

[28] Smith ER (2014). Serum calcification propensity predicts all-cause mortality in predialysis CKD. J. Am. Soc. Nephrol..

[29] Dahle DO (2016). Serum calcification propensity is a strong and independent determinant of cardiac and all-cause mortality in kidney transplant recipients. Am. J. Transpl..

[30] Pruijm M (2017). Serum calcification propensity is associated with renal tissue oxygenation and resistive index in patients with arterial hypertension or chronic kidney disease. J. Hypertens..

[31] Allison MA (2012). Calcified atherosclerosis in different vascular beds and the risk of mortality. Arterioscler. Thromb. Vasc. Biol..

[32] Pasch A (2012). Nanoparticle-based test measures overall propensity for calcification in serum. J. Am. Soc. Nephrol..

[33] Miura Y (2018). Identification and quantification of plasma calciprotein particles with distinct physical properties in patients with chronic kidney disease. Sci. Rep..

[34] Wu CY, Young L, Young D, Martel J, Young JD (2013). Bions: a family of biomimetic mineralo-organic complexes derived from biological fluids. PLoS one.

[35] Peng HH (2017). Mineral particles stimulate innate immunity through neutrophil extracellular traps containing HMGB1. Sci. Rep..

