# Supplementary material for: Ectopic calcification and formation of mineralo-organic particles in arteries of diabetic subjects
Source: Sci Rep. 2020 May 22;10:8545. doi: 10.1038/s41598-020-65276-7 (PMC7244712; doi:10.1038/s41598-020-65276-7)
Supplement: Supplementary file 1 — Supplementary Information. [file 41598_2020_65276_MOESM1_ESM.pdf]

Supplementary Information

Ectopic calcification and formation of mineralo-organic particles in arteries of diabetic subjects

Cheng-Yeu Wu[1,2,3], Jan Martel[1,2,4], and John D. Young[1,2,4,5]

[1]Laboratory of Nanomaterials, Chang Gung University, Taoyuan, 33302, Taiwan

[2]Center for Molecular and Clinical Immunology, Chang Gung University, Taoyuan, 33302, Taiwan

[3]Research Center of Bacterial Pathogenesis, Chang Gung University, Taoyuan, 33302, Taiwan

[4]Chang Gung Immunology Consortium, Linkou Chang Gung Memorial Hospital, Taoyuan, 33305, Taiwan

[5]Biochemical Engineering Research Center, Ming Chi University of Technology, New Taipei City, 24301, Taiwan

Supplementary Information

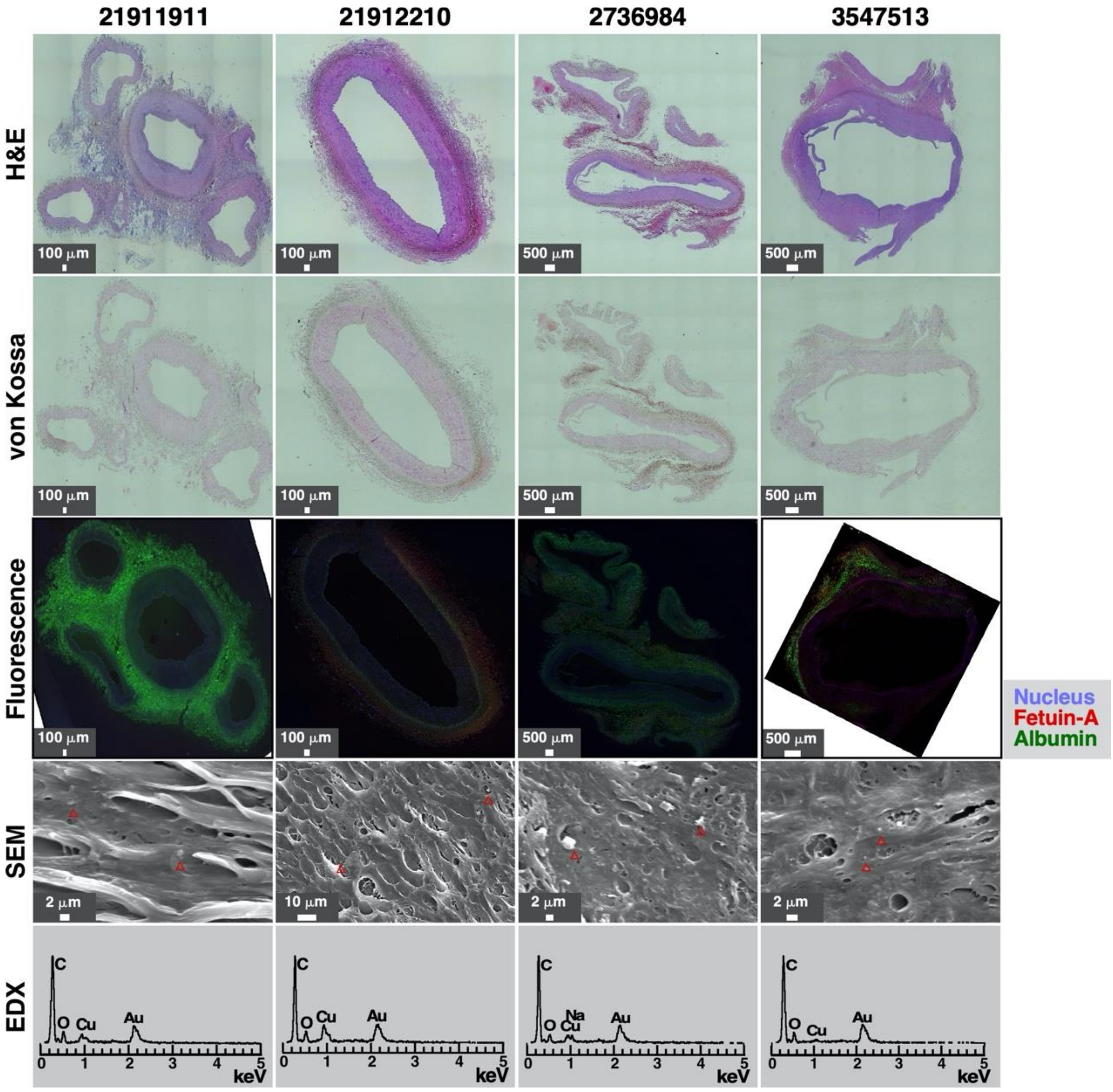

Supplementary Figure S1. Microscopy and spectroscopy analyses of arteries from healthy individuals. Lower limb arteries of healthy controls were prepared for histology and microscopy analyses as described in *Methods*. No calcification was detected in these samples stained with von Kossa. While some NPs were observed under SEM, calcium and phosphorus were not detected by EDX in these particles.

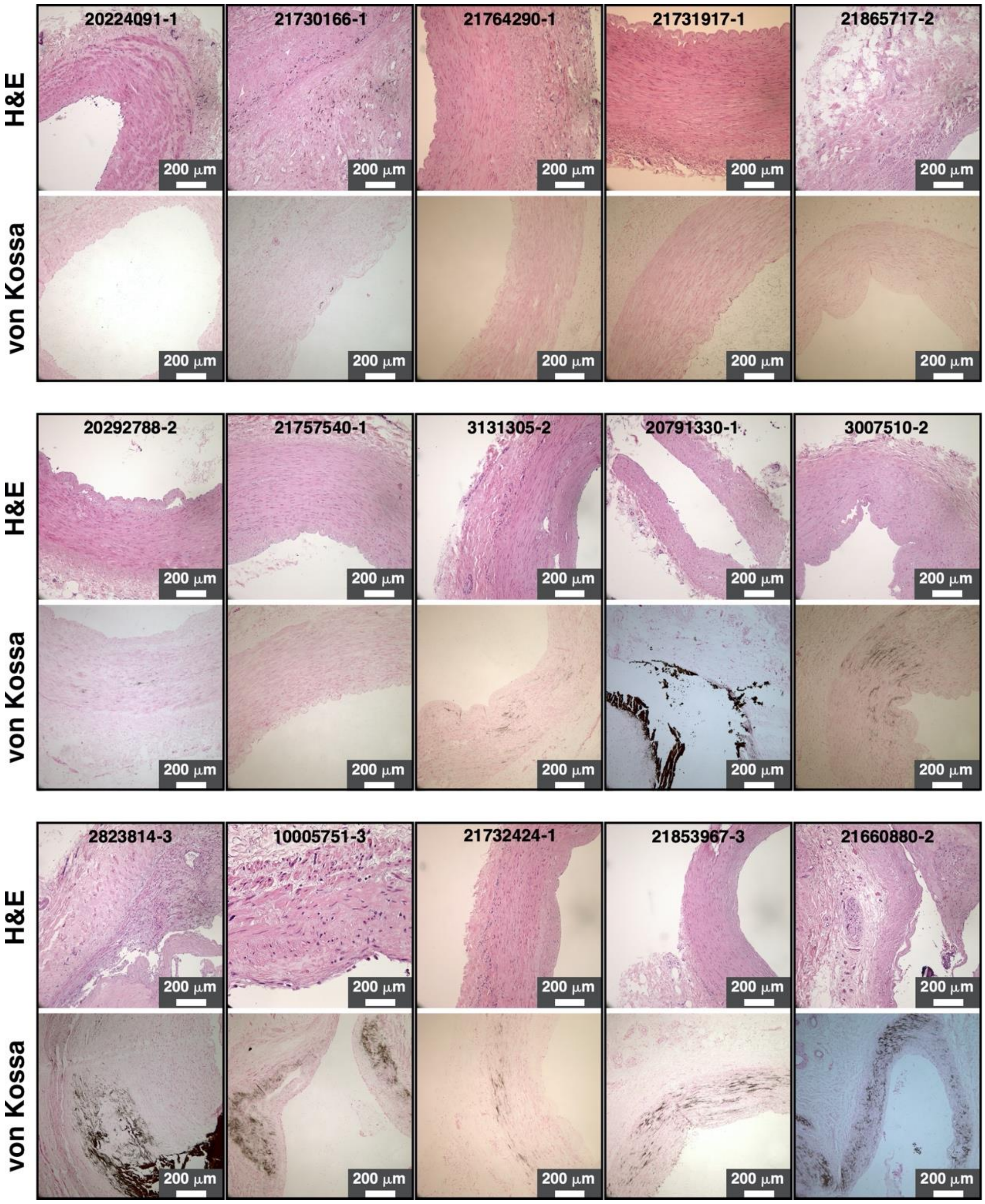
H&E
von Kossa
20224091-1
21730166-1
21764290-1
21731917-1
21865717-2
20292788-2
21757540-1
3131305-2
20791330-1
3007510-2
2823814-3
10005751-3
21732424-1
21853967-3
21660880-2
200 μm

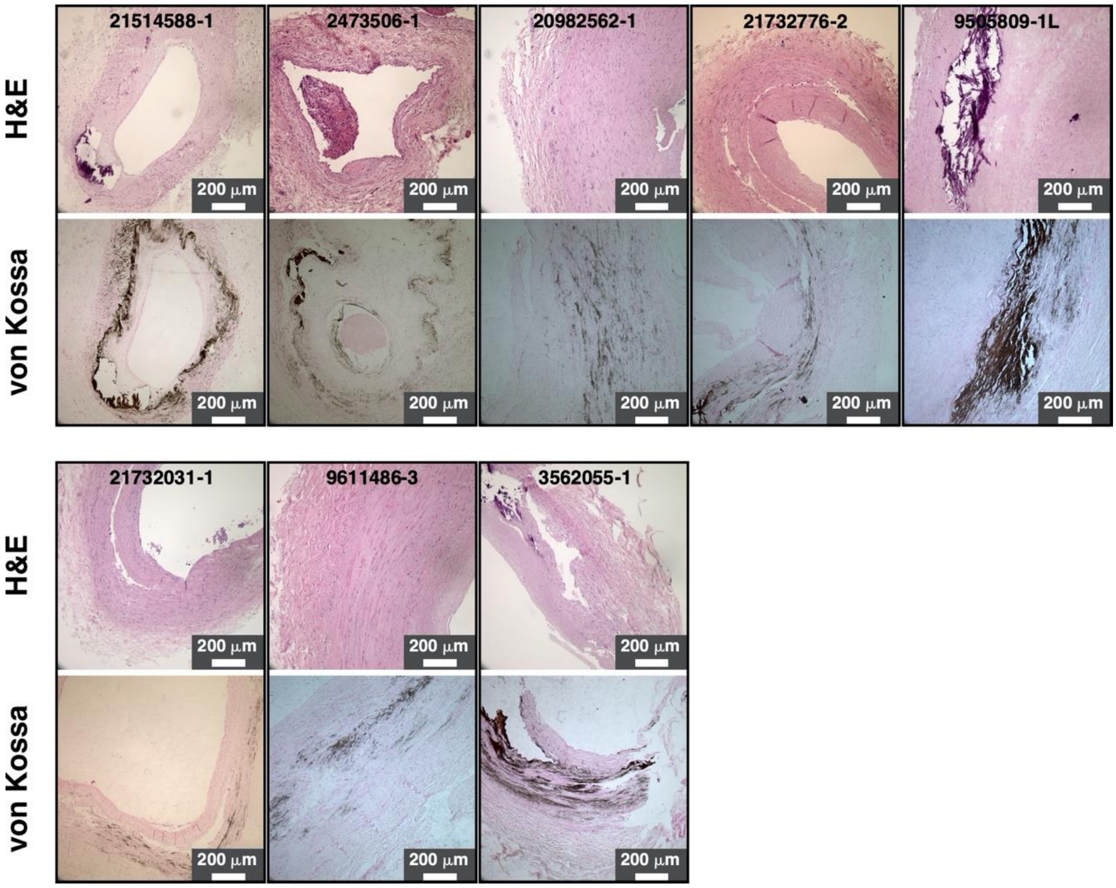

Supplementary Figure S2. H&E and von Kossa staining of lower limb arteries from diabetic individuals. Tissues were processed as described in the *Methods*.

Supplementary Table S1. Calcification level and artery condition of the subjects examined in the present study

	Sample #	Calcification Level	Intimal Thickening	Intimal Calcification	Condition
1	21911911	Not detected	–	–	Healthy individual
2	21912210	Not detected	–	–	Healthy individual
3	2736984	Not detected	–	–	Healthy individual
4	3545713	Not detected	–	–	Healthy individual
5	20224091-1	Stage 0	–	–	Type 2 diabetes
6	21730166-1	Stage 0	–	–	Type 2 diabetes
7	21764290-1	Stage 0	–	–	Type 2 diabetes
8	21731917-1	Stage 0	–	–	Type 2 diabetes
9	21865717-2	Stage 1	–	–	Type 2 diabetes
10	20292788-2	Stage 1	–	–	Type 2 diabetes
11	21757540-1	Stage 1	+	–	Type 2 diabetes
12	3131305-2	Stage 2	+	–	Type 2 diabetes
13	20791330-1	Stage 2	+	+	Type 2 diabetes
14	3007510-2	Stage 2	+	–	Type 2 diabetes
15	2823814-3	Stage 2	+	+	Type 2 diabetes
16	10005751-3	Stage 2	+	+	Type 2 diabetes
17	21732424-1	Stage 2	+	–	Type 2 diabetes
18	21853967-3	Stage 3	+	–	Type 2 diabetes
29	21660880-2	Stage 3	+	+	Type 2 diabetes
20	21514588-1	Stage 3	+	–	Type 2 diabetes
21	2473506-1	Stage 3	+	+	Type 2 diabetes
22	20982562-1	Stage 3	+	–	Type 2 diabetes
23	21732776-2	Stage 3	+	+	Type 2 diabetes
24	9505809-1L	Stage 3	+	+	Type 2 diabetes
25	21732031-1	Stage 3	+	–	Type 2 diabetes
26	9611486-3	Stage 3	+	–	Type 2 diabetes
27	3562055-1	Stage 3	+	+	Type 2 diabetes
